# Art therapy-based interventions to address burnout and psychosocial distress in healthcare workers—a systematic review

**DOI:** 10.1186/s12913-023-09958-8

**Published:** 2023-10-04

**Authors:** Megan Tjasink, Eleanor Keiller, Madison Stephens, Catherine Elizabeth Carr, Stefan Priebe

**Affiliations:** Queen Mary University of London, London, UK

**Keywords:** Art therapy, Healthcare worker, Burnout, Psychosocial distress, Anxiety, Stress

## Abstract

**Background:**

Burnout and psychosocial distress are serious and growing issues for healthcare workers (HCWs) and healthcare systems across the globe. Exacerbated by changes in healthcare delivery during and following the Covid-19 pandemic, these issues negatively affect HCW wellbeing, clinical outcomes and patient safety. Art Therapy has demonstrated promise as a suitable but under researched intervention, warranting further investigation. This systematic review aims to ascertain what art therapy-based interventions used to address burnout and / or psychosocial distress in HCWs have been reported in the health and social care literature and how these have been evaluated.

**Methods:**

Six databases (PubMed, PsycINFO, MEDLINE, EMBASE, CINAHL, ProQuest Central), Google Scholar and three clinical trial registries (CENTRAL, ICTRP and ClinicalTrials.gov) were searched for studies using art therapy-based methods to engage with burnout risk or psychosocial distress in HCWs. Following screening for eligibility study characteristics and outcomes were extracted by two reviewers independently. Studies were evaluated using the Joanna Briggs Institute (JBI) Critical Appraisal Tools. Outcomes were grouped for analysis. Quantitative and qualitative results were synthesised and integrated using narrative synthesis.

**Results:**

Twenty-seven studies, drawn from thirteen countries, spanning five continents were selected for inclusion. Fifty percent were published in the last five years, indicating growing global research in the field. Fourteen studies used quantitative research methods and thirteen used qualitative methods. A total of 1580 participants took part in the studies, with nurses most broadly represented (59%). Interventions were mostly delivered in groups (95%) and by an art therapist (70%). Heterogeneity and insufficient randomised controlled trials precluded the possibility of meta-analysis. However, a review of available data showed evidence of medium to large effects for emotional exhaustion (burnout), work-related stress and common mental health issues. A content analysis of qualitative data of perceived effect complemented quantitative findings.

**Conclusion:**

Global research into the use of art therapy-based methods to address burnout and psychosocial distress in HCWs is growing. Whilst further high-quality evidence such as randomised controlled trials would be beneficial, findings suggest that art therapy-based methods should be strongly considered as an acceptable and effective treatment for symptoms of emotional exhaustion (burnout) and psychosocial distress in HCWs.

**Supplementary Information:**

The online version contains supplementary material available at 10.1186/s12913-023-09958-8.

## Introduction

Burnout in healthcare workers (HCWs) is a serious and growing global issue. Caused by exposure over time to chronic work-related stressors [1] such as overly burdensome workloads, long working hours, under-resourcing and work-place conflict [2], burnout has a negative impact, not only on individuals, but on healthcare systems. It can result in a reduction in time and effort at work [3], which can lead to poor clinical outcomes and staff shortages that may impact on patient safety [4]. Burnout also places a large financial burden on already over-stretched healthcare systems. Prior to the Covid-19 pandemic a conservative estimate of physician burnout alone in the United States (US) was $4.6 billion a year [5]. Mental distress such as depression and suicidal ideation is strongly associated with burnout in HCWs [6, 7] and similarly impacts on staffing levels and the delivery of patient care. In the United Kingdom (UK), one quarter of sickness absence for nurses is due to anxiety, stress, or depression [8], and increases in HCW burnout following the pandemic have been reported globally [9, 10]. There is concern within the medical field that complacency to address burnout in HCWs now will endanger the future global supply of quality healthcare, having “profound consequences for billions of people around the world” [11]. Whilst much has been written about the issue and implications of burnout and increased psycho-social distress in healthcare professionals [12, 13], research into effective interventions to address these is in early stages [14].

Prior to and during the Covid-19 pandemic, art therapy-based interventions (in which art therapists and other trained healthcare professionals used visual art methods for the purpose of psychosocial support) showed promise as a means of alleviating symptoms of burnout and psychosocial distress in healthcare workers [15–18]. The Covid-19 pandemic prompted new urgency in relation to staff support with calls to reconfigure services [19]. It is important at this juncture to understand the potential role of art therapy methods in helping to address this serious issue faced by healthcare professionals and providers. Objectives of this systematic review of the literature were: To elicit what art therapy-based interventions used to address burnout and / or psychosocial distress in healthcare workers have been reported in the health and social care literature and how the effect of these interventions has been evaluated. To our knowledge there has not been a previous systematic review on the topic, and a better understanding of these factors could help to inform service-level decision-making and help focus future research.

## Methods

### Study design

The Preferred Reporting Items for Systematic Reviews and Meta-Analysis (PRISMA) recommendations were utilized for conducting this systematic review [20]. The review protocol was registered with PROSPERO, the international prospective register of systematic reviews on the 8^th^ of March 2022 (registration code: CRD42022313788).

### Search strategy and data sources

The following electronic databases were systematically searched following PRISMA guidelines: PubMed, PsycINFO, MEDLINE, EMBASE, CINAHL, ProQuest Central, Google Scholar and web based clinical trial registries including CENTRAL, ICTRP and ClinicalTrials.gov. There was no date restriction in database searches. The search strategy included a combination of key words for population, outcome, and intervention. These were adjusted when necessary, according to requirements of each database. Following the completion of electronic database searches, the following three art therapy journals with the greatest impact factor in the field were hand searched: “International Journal of Art Therapy”, “Art Therapy, The Journal of the American Association of Art Therapy” and “Arts in Psychotherapy”. Hand searches of these journals were restricted to the past 20 years. A snowballing technique was then used to find any further studies from the bibliographies of relevant literature returned. The reviewers made personal contact with authors for further information where necessary. There were no language restrictions.

### Selection criteria

#### Study type

Randomised controlled trials, non-randomised controlled studies, non-controlled quasi-experimental studies and non-experimental studies such as case studies were included. All study designs including those using multimodal methods were eligible for inclusion. Unpublished studies were included. Literature reviews, editorials, book chapters, PhD theses and commentaries were excluded.

#### Condition

Professional burnout, work-related stress, psychological and psychosocial distress in healthcare workers were included. Professional development or primarily educational outcomes (such as developing practice skills) were excluded.

#### Population

Adults working as healthcare professionals in patient-facing roles, such as nurses, doctors, medical social workers, and allied health professionals were included. Trainee nurses and doctors engaged in patient-facing work were also included. Informal carers were excluded.

#### Intervention

Art Therapy is a psychological therapy using art making and creative expression through visual art media as a key element of treatment. For the purposes of this review any therapeutic intervention using visual art as a key element of treatment for burnout or psychosocial distress was included. Any visual art form (such as painting, drawing, collage and clay modelling) used therapeutically by a trained health or social care professional was considered for inclusion. Art making or art viewing primarily for recreational purposes was not included. Art making facilitated primarily by artists or non-clinician educators was excluded. The broader arts such as drama, music and dance were excluded unless the study also used visual art as a primary element of treatment. Any discrepancies regarding inclusion or exclusion in relation to these criteria were discussed by the review team until a consensus was reached.

#### Outcomes

Quantitative and qualitative outcomes including participant experiences and practitioner observations were reported. A range of instruments used to assess changes in behaviour, psychological and psychosocial factors in relation to burnout and psychosocial distress were included. Instruments primarily measuring personal or professional development were not included. Qualitative outcomes included data collected through interviews, open-ended questionnaires therapist reports and participant output including visual artwork and group discussion.

Search terms used for the review are presented in Table 1:

**Table 1:** Search terms

Burnout OR mental distress OR professional burnout OR compassion fatigue OR personal accomplishment OR resilience OR stress OR emotional exhaustion OR depersonalization OR mental health OR depression OR anxiety AND	Art therap* OR art psychotherap* OR art-based intervention* OR creative art* therap* OR expressive art* therap* OR mandala OR painting therapy OR drawing therapy OR clay therapy OR visual art therap* AND	Anaesthetist* OR clinician* OR nurs* OR doctor* OR physician* OR medical professional* OR hospital staff OR medical student* OR healthcare professional*OR surgeon*

Database specific truncation and operators were used. Database and hand searches were completed on 25 March 2022.

### Data collection

Systematic review management software Covidence was used for independent screening and data extraction. Studies found via database and hand searches were uploaded to Covidence and titles and abstracts were screened by the first reviewer for eligibility. A proportion of studies selected were checked by a second reviewer and any discrepancies at this stage were resolved through discussion, with input from a third senior reviewer if necessary. Agreed full texts were then retrieved. These were screened independently by two reviewers for eligibility.

Data extraction was performed by two independent members of the review team using an adapted version of the Covidence data extraction form. Data extraction included study identifiers: methods, population, intervention characteristics and outcomes. Mean quantitative outcomes and standard deviation for two time points, pre and post intervention were extracted where available. Authors were contacted via email for unreported data. Qualitative data were extracted in relation to the following categories pre-defined on the data extraction form: Participant reported experience of perceived effect, practitioner reported observations of perceived effect, and practitioner / participant report of perceived risk or harm.

### Data synthesis

The characteristics of each study were summarised as per the predefined criteria. Findings were tabulated and studies were grouped according to study design, outcome measure and intervention characteristics. These groupings were used to assess heterogeneity to determine whether it would be possible to undertake a meta-analysis in addition to a narrative synthesis based on the synthesis without meta-analysis reporting items (SWiM) checklist [21]. Content analysis was applied to qualitative outcomes extracted by two reviewers independently using a pre-defined data extraction form. Line by line coding was used to define key themes. Themes were derived from codes appearing in three or more studies. Content analysis was reported as recommended in ENTREQ guidelines [22].

Tables were synthesised using vote counting based on direction of effect. These results were reported using a Harvest plot, along with a description of any available effect estimates, and the limitations of this methodology. The quantitative and qualitative syntheses were integrated using narrative summary. Joanna Briggs (JBI) recommendations for mixed methods syntheses were followed.

### Quality assessment

Two members of the review team independently appraised risk of bias of intervention effects / study quality using Joanna Briggs Institute (JBI) Critical Appraisal Tools [23]. Studies were allocated to one of four groups (RCT, quasi-experimental, qualitative, or case study) to compare and discuss. The JBI tools were chosen due to the range of study designs included in this review. Subgroups of studies were additionally reviewed for certainty of evidence in relation to outcome categories using the GRADEPro Guideline Development Tool (GDT) [24].

## Results

### Identification and selection of studies

Database and hand searches yielded 5481 titles. Following the searches, 561 duplicates were removed. 4920 titles and abstracts were screened for relevancy according to the inclusion and exclusion criteria identified prior to the search. 95 full texts were retrieved. These were screened independently by two reviewers for eligibility. 68 studies were excluded, leaving 27 studies for inclusion in the systematic review.

**Fig. 1:**
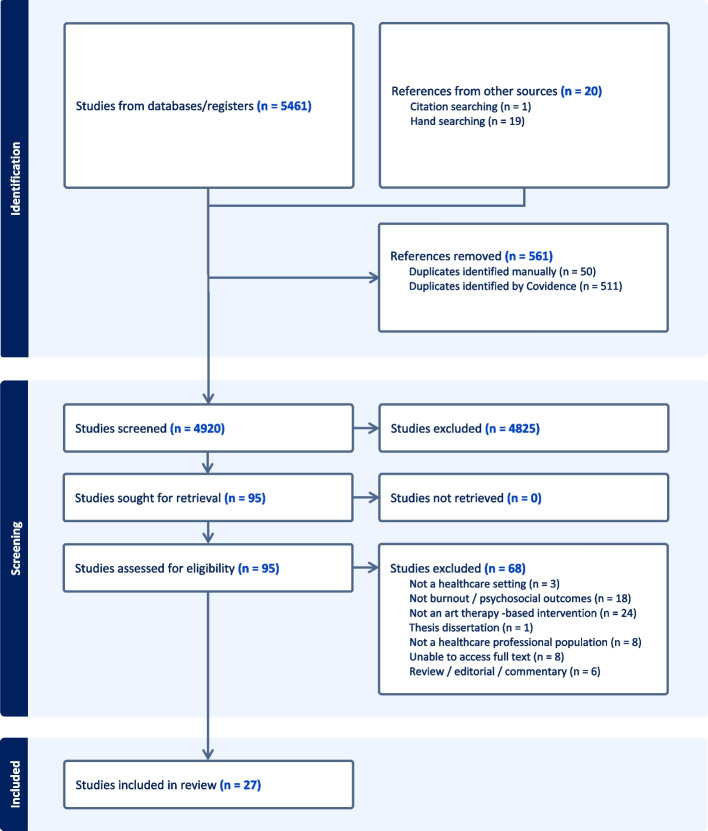
PRISMA flowchart of study selection process

Agreement between reviewers was 86% This was calculated to be a substantial agreement of 0.69 using Cohen’s Kappa. Discrepancies were resolved through discussion and input from a third senior reviewer. The Preferred Reporting Items for Systematic Reviews and Meta-Analyses (PRISMA) process was employed to ensure transparent reporting (Fig. 1).

### Study characteristics

The studies included in this review took place in thirteen countries, spanning five continents. The largest number of publications were from the US (8), followed by the UK (4) and Hong Kong (3). Most studies took place in high-income countries, with three from upper middle-income countries and none from lower middle- or low-income countries (as defined by the World Bank Classification, 2022 [25]). The studies were published between 1994 and 2022, with 50% published in the last 5 years, indicating a recent increase in studies on the use of art therapy-based methods with healthcare workers.

Fourteen of the included studies used quantitative research methods, collecting pre- and post-intervention data via a range of validated and non-validated outcomes measures. Six of these studies included quantitative measures only, and eight employed mixed methods—collecting both quantitative and qualitative data. Four studies had a randomised comparative condition. However, only one of these was a true randomised control trial [17] whilst the other three were pilot / preliminary studies. A further four quasi-experimental studies had a non-randomised control group, and six were non-controlled studies.

Of the remaining thirteen studies, five were qualitative: collecting data via a range of qualitative enquiry methods, including semi-structured interviews, participant surveys and content analysis of visual, verbal, or written participant output. Eight studies were case reports. Most of the case reports employed qualitative and/ or quantitative evaluation.

Studies were classified and grouped for analysis by two independent reviewers with input from a third senior reviewer.

### Condition

Professional burnout and several conditions or states falling under the umbrella of ‘psychosocial distress’ were included. These were grouped into the following six categories for analysis: Burnout, anxiety, stress, emotional state, trauma, and quality of life. Whilst there may be a relationship between the two, conditions relating primarily to self- or professional-development such as: Professional- and self-efficacy were excluded.

### Population

**Fig. 2:**
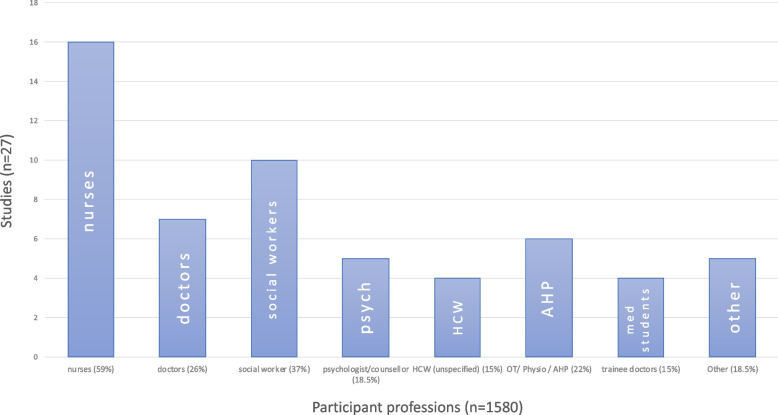
Participant job roles. Figure 2 key: Nurses, Doctors, Social Workers, Psychologists / Counsellors, Health Care Workers (unspecified), Allied Health Professionals (including Occupational Therapists and Physiotherapists), Medical Students, Other (including trainee nurses, patient care technicians, healthcare assistants, hospital clergy, professional carers)

A total of 1580 participants took part in the studies included in this review. Due to missing demographic data, some population characteristics could not be fully described. The job roles of participants were specified in most studies, and a range of healthcare professionals were included. Nurses were most represented, named in 59% of studies, followed by social workers (37%) and doctors (26%). Patient-facing medical students were included in 15% of studies (Fig. 2). Participants were drawn from a range of clinical areas with hospice / end of life and general hospital settings most represented in 30% and 26% of studies respectively.

Nineteen studies (*n* = 857 accounting for missing data on 7 participants) reported gender. Of these, 83% of participants were female and 17% male. No non-binary participants were reported. Seventeen studies (*n* = 992) reported the age of participants. Of these studies, the age range was 18 – 69. Only six studies provided the mean age of their cohorts. These varied from 43.5 years (in a population of homecare hospice healthcare workers in Singapore [17]) to 23 years in a population of newly hired cancer nurses in Korea [26]. Only five studies reported the ethnicity of participants (*n* = 407). Of the participants included in this small sample, 56.5% were Asian, 38% Caucasian, 2.5% Hispanic, 0.2% African American and 3.2% other.

### Intervention

**Fig. 3:**
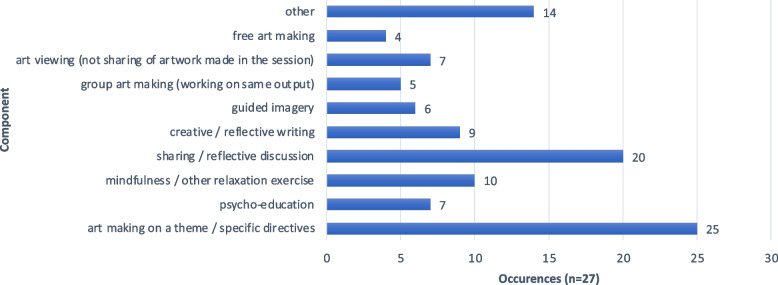
Intervention components across studies

A range of interventions using art as a key element of treatment for burnout or psychosocial distress were included. Intervention characteristics are summarised in Table 2. 70% were provided by or with an art therapist (art therapists (52%), art therapists alongside another professional such as a mindfulness practitioner or a psychologist (17%)), and 55% of interventions included “art therapy” or “art therapy-based …”, in the intervention name, with a further 7% including creative or expressive arts therapies. Other interventions included “mandala-making” (11%) and several less frequently occurring interventions such as “art-based debrief” and “response art” (both involving artmaking as a way of processing clinical material) and “Zentangle art” (a meditative form of drawing using repetitive patterns). Interventions were most provided in group format (95%) with the most frequent group size consisting between 2 to 8 members. Single sessions and short blocks of 2 – 4 sessions made up the majority (62%) of interventions. Whilst more than half of studies did not specify where the intervention took place, spaces within hospitals and academic institutions were most cited. Intervention components reported in studies (summarised in Fig. 3) usually included art making on a theme or with specific directives (featuring in 93%) and sharing or reflective discussion (74%). Free, non-directed art making was employed in 15% of interventions. More than half of interventions (64%) included non-art therapy-specific elements such as Mindfulness or relaxation exercises (37%), creative or reflective writing (33%) and psychoeducation (26%). (Percentage calculations account for unreported data.) Two studies ( [16] and [26]) included other arts therapies (dance movement therapy and music therapy). In Moss [14] participants could choose which form of creative arts therapy they wished to engage with. Only one study [27] reported the use of an intervention adherence tool.

**Table 2:** Summary of interventions

Name of intervention (*n* = 27)	Who provided?	How provided?	How much?	Where?
Art Therapy **(10)**	Art Therapist **(12)**	Group 2–8 **(9)**	Single session < 2 h **(3)**	Hospital** (4)**
Art therapy – based groups **(3)**	Art Therapist with other (curator, mindfulness practitioner, psychologist, social worker) **(4)**	Group 9 – 12** (5)**	Single session ≥ 2 h **(5)**	Academic institution **(4)**
Mandala making **(3)**	University faculty member (medicine or nursing) **(2)**	Group > 12 **(6)**	2 – 4 sessions **(8)**	Hospice** (3)**
Art-based debrief **(2)**	Psychologist / psychological therapist **(2)**	Individual **(1)**	5 – 8 sessions **(6)**	Art gallery **(1)**
Creative / Expressive Arts Therapy **(2)**	Nurse** /** CNS** (2)**		> 8 sessions **(4)**	Retreat Centre **(1)**
Mindful-Compassion Art-Based Therapy (MCAT) **(1)**	Certified Zen-tangle Teachers (CZT®) **(1)**			
Collaborative Art making **(1)**				
Response Art **(1)**				
Zentangle Art** (1)**				
Visual journaling **(1)**				
Arts-based resiliency curriculum **(1)**				
Nurse-led intervention **(1)**	Not specified **(4)**	Not specified **(6)**	Not specified** (1)**	Not specified **(14)**

Interventions were summarised using the Template for intervention description and replication (TIDieR) checklist [28].

Intervention components are summarised in Fig. 3. Art making on a theme or with specific directives was the most common component, featuring in 93% of studies, followed by sharing / reflective discussion reported in 74% of studies. The “other” category included elements such as visual journaling, goal setting, and participants being given art materials to use at home in their own time.

### Outcomes

The fourteen quantitative studies in this review used twenty-eight different outcomes measures. Nineteen of these measures met the inclusion criteria (measuring burnout or psychosocial distress).

The thirteen studies collecting qualitative outcomes used a range of data gathering methods, with some using more than one method. Five studies used an evaluation survey post intervention, one study used a pre- and post-intervention questionnaire, four used semi-structured interviews, four used participant output (creative writing and / or artwork) and three used therapist / researcher field notes or audio recording of sessions. Fourteen codes for intervention effects were derived from a content analysis of extracted qualitative data. 

### Quality appraisal

**Fig. 4:**
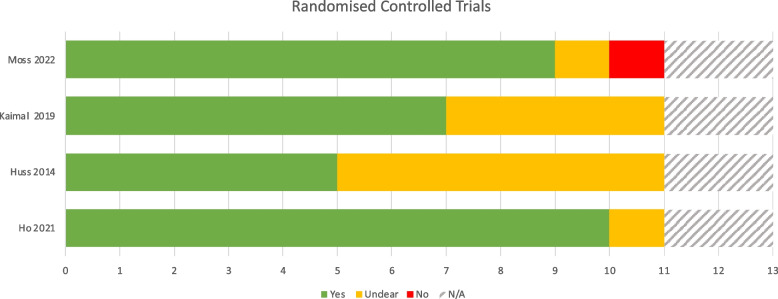
JBI RCT checklist results

**Fig. 5:**
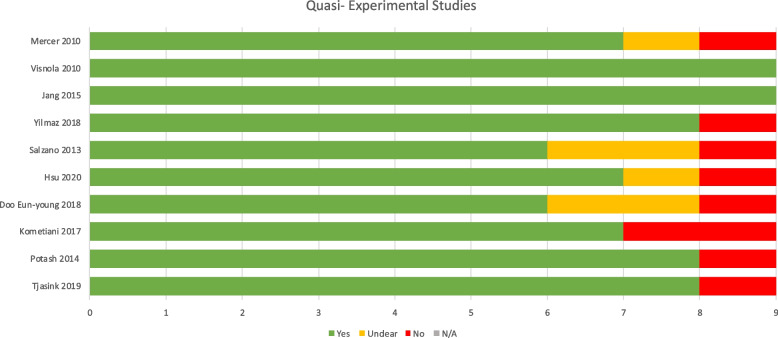
JBI Quasi-experimental checklist results

**Fig. 6:**
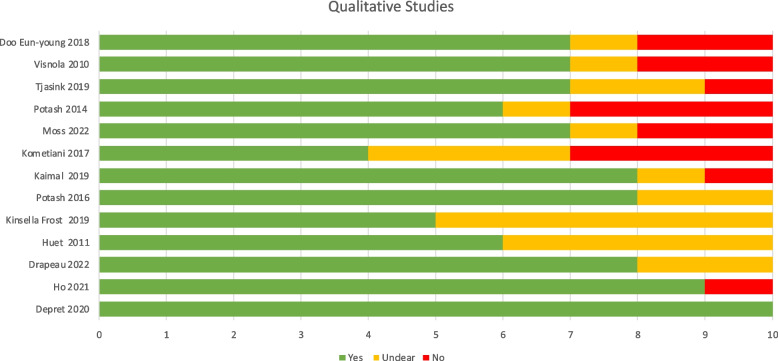
JBI qualitative studies checklist results

**Fig. 7:**
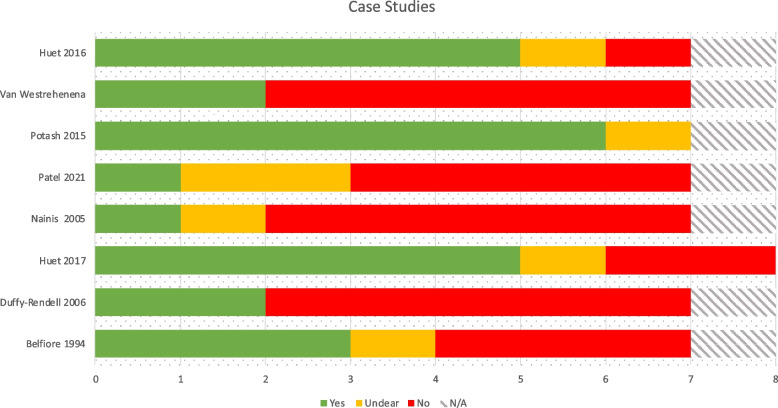
JBI case studies checklist results

Results of quality appraisal are reported in Figs. 4, 5, 6 and 7 according to study type using the JBI Critical Appraisal Tools. Each tool comprises a series of questions regarding methodological rigour relevant to the study type. These are answered as “Yes”, No” or “Unclear” by two independent reviewers. Studies with more “Yes” answers are appraised as methodologically more rigorous. Whilst studies varied in methodological rigour, none were excluded.

#### Quantitative studies

The four studies using randomised comparison or control groups were all mixed methods and were appraised against both the RCT and Qualitative studies checklists. In relation to the RCT checklist, all used outcomes measures in a reliable way and used appropriate statistical analysis methods. However, analysis was limited in the two smaller pilot studies reportedly because of their preliminary nature. Control and treatment groups were similar at baseline in all but one study which accounted for baseline differences in the analysis. However, it was unclear due to lack of reporting whether true randomization was used for assignment of participants to treatment groups in two of the pilot studies. Deviations from standard RCT design were deemed to not be fully accounted for in the two smaller pilot studies.

Due to the nature of behavioural studies neither participants nor those delivering the intervention could be blinded to treatment group assignment, so these two questions were considered not applicable. Blinding of outcomes assessors was unclear due to lack of reporting in three of the studies, whilst one study [17], reported unblinded outcomes assessors. Discounting the two non-applicable questions, studies range from 45.5% to 90.9% affirmative responses on the JBI checklist for RCT’s, with and average score of 70.4%. Whilst the JBI does not provide fixed graded categories, this could be interpreted to represent moderate to low risk of bias overall despite the inclusion of preliminary studies. The true RCT (Ho [17]) and the largest pilot (Moss [16]) scored highly on the checklist and were thus deemed to be low risk of bias.

The quasi-experimental (non-randomised) studies scored highly against the relevant JBI checklist, with an affirmative response range of 66.7% to 100% and an average of 83.3%. The most common introduction of bias was the lack of a control group, evident in 5 out of 10 studies. This was followed by issues with missing follow up data, with three studies deemed to have incomplete or inadequately described or analysed follow up data. Strengths included clarity of cause and effect, the use of multiple (pre and post) measures and the reliable use of outcomes measures. The three mixed methods quasi-experimental studies [29–31] were also checked against the qualitative studies tool.

#### Qualitative studies

The thirteen studies checked against the JBI qualitative research checklist varied in quality from high to low risk of bias, ranging from 40% to 100% affirmative responses with an average of 70.8%. There were a number of methodological weaknesses seen across the qualitative studies. For example, only five studies included a clearly stated philosophical perspective, congruous with the research methodology. Similarly, only five included a statement locating the researcher culturally or theoretically. Even fewer studies ([17, 32, 33]) addressed the influence of the researcher on the research. Participants, and their voices, were deemed to be inadequately represented in nearly half of studies, with a tendency towards reporting the therapist / researcher’s opinion of participant experience and lack of direct quotes from participants. There are examples to the contrary, such as thematic analysis of participant feedback supported by direct quotes in Tjasink et al. [29]. Where participants’ artwork was included in studies, this was generally used to illustrate points made by the author, and participants’ own accounts of their artwork were poorly represented. Exceptions included Kaimal et al. [18], who included participants’ (verbatim) experiences alongside their artwork.

Strengths of the studies included congruity between the research methodology and the methods used to collect data and a relatively high level of congruity between the research methodology and the research objectives. Most conclusions drawn flowed from the analysis, or interpretation of the data and most studies included a statement regarding ethical approval of the research.

#### Case studies

Included case studies represented a broad spectrum in relation to methodological rigour. Despite a large range of 14.3% to 85.7% affirmative answers against the JBI checklist, none were excluded as an aim of the review was to understand breadth of practice. Considered as a group, the case reports had an average of 45.3% affirmative answers when adjusted for a non-applicable question. This pointed to widespread methodological flaws and suggested serious risk of bias. Areas of potential bias included a lack of clarity in relation to assessment methods, with only one study deemed to meet this criterion. Similarly, lack of reporting in relation to adverse or unanticipated events, (only two studies acknowledged these) was a potential indicator of bias. Only two studies clearly described the current clinical condition of participants, and three of the eight clearly described participants’ post intervention condition. This could be due to participants being professionals rather than patients with diagnoses. However, as interventions were aimed at improving psychosocial factors in participants, the authors considered questions about participants’ conditions pre and post intervention relevant and retained these as part of the critical appraisal. Another potential reason for the general lack of clarity regarding participants is that case studies all reported group-based interventions. To mitigate this, the authors agreed the checklist question: ‘Was the patient’s history clearly described and presented as a timeline?’ to be not applicable, although this criterion was addressed in one study [34] which provided background information for each member of a small group. Participants’ demographic characteristics were clearly described in less than half the studies. There were some strengths across the case reports too, with all but one providing a takeaway lesson and more than 50% of studies clearly describing the intervention. It should be noted that the JBI critical appraisal tools include a ‘case series’ checklist, but although some studies described groups with multiple and sequential cohorts, none used a standard case series study design. Reasons for this were not clear but having done so may have facilitated better reporting and methodological rigour.

Overall a lack of methodological rigour in relation to study design and unclear or incomplete reporting, seen particularly in qualitative studies and case reports, impacted on the quality of evidence. In the quantitative studies, small to modest sample sizes and the low number of randomised controls increased risk of bias. However, despite these issues, exemplary studies stood out as examples of well-conceived, high-quality research across all categories.

### Synthesised quantitative findings

Due to the large number of different outcomes measures used, these were grouped for synthesis according to the condition they measured. Five categories: Burnout, Anxiety, Stress, Emotional State, Trauma and Quality of Life are summarised in Table 3. The most frequently measured condition was burnout, featuring in just over half of quantitative studies. Whilst largely homogenous outcomes were used to measure burnout, this was not the case across other conditions. The most heterogeneous grouping was stress, with nine different tools, including validated and unvalidated measures and biomarkers, used across five studies.

**Table 3:** Summary of conditions and outcomes measures

Condition	Number of studies measuring condition	Number of outcomes measures used	Outcomes measures used
Burnout	8	2	Seven 16-item MBI, one abridged MBI
Anxiety	4	3	One HADS, three STAI, 1 short version of the (PROMIS®) tool for anxiety in adults
Stress	5	8	Five different job stress tools, two cortisol (saliva), one ILP (saliva), one CRP (saliva)
Trauma	2	2	One PDS-5, one PTGI
Emotional state	5	3	Three PANAS, one SUDS, one BSRS-5
Quality of Life	5	2	Four ProQOL, one EUROHIS-QoL -8

Effect sizes were estimated for intervention and control / comparison groups using Cohen’s *d* where data were available. Authors were contacted for further information where mean pre-post scores and / or measures of variation were not reported, however no further data were obtained in this manner. Where possible, only data relating to the art therapy element were extracted when art therapy was delivered alongside other creative arts therapies.^1^

Estimated effect sizes seen in Table 4 can be interpreted using Cohen’s [35] guidelines of small (0.2), medium (0.5) and large (0.8) effect.

**Table 4:**
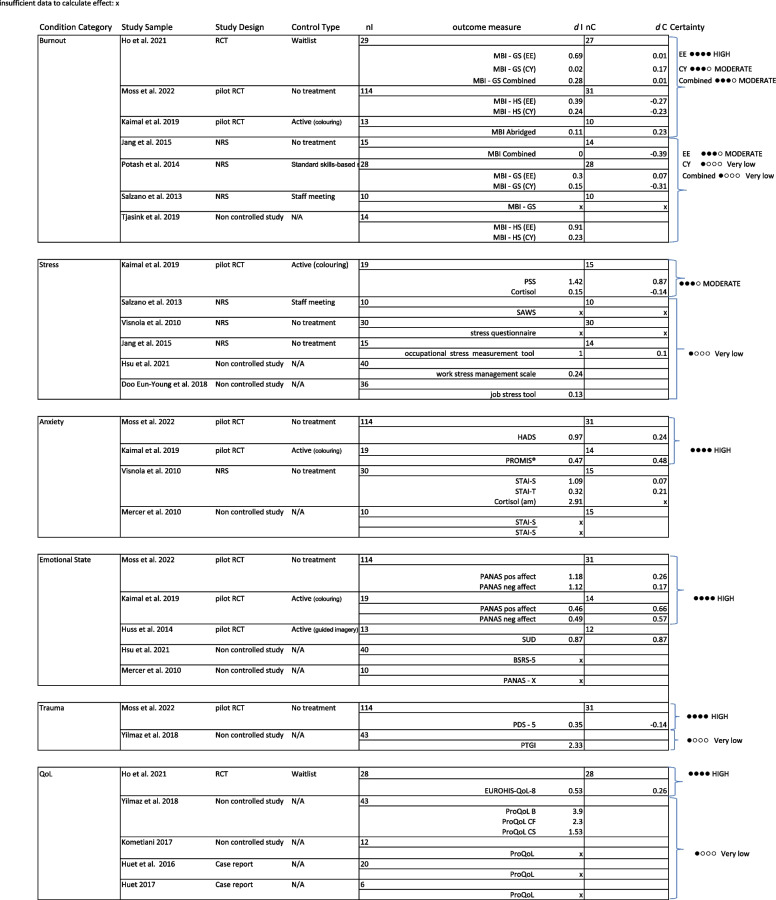
Estimated effect sizes and certainty of evidence by outcome category

Categories: “burnout”, “anxiety” and “emotional state” were more frequently represented in study designs which provided higher quality evidence, based on the GRADE system [36]. Studies measuring these outcomes were additionally deemed to not have significant risk of bias (Figs. 4, 5, 6 and 7).

**Fig. 8:**
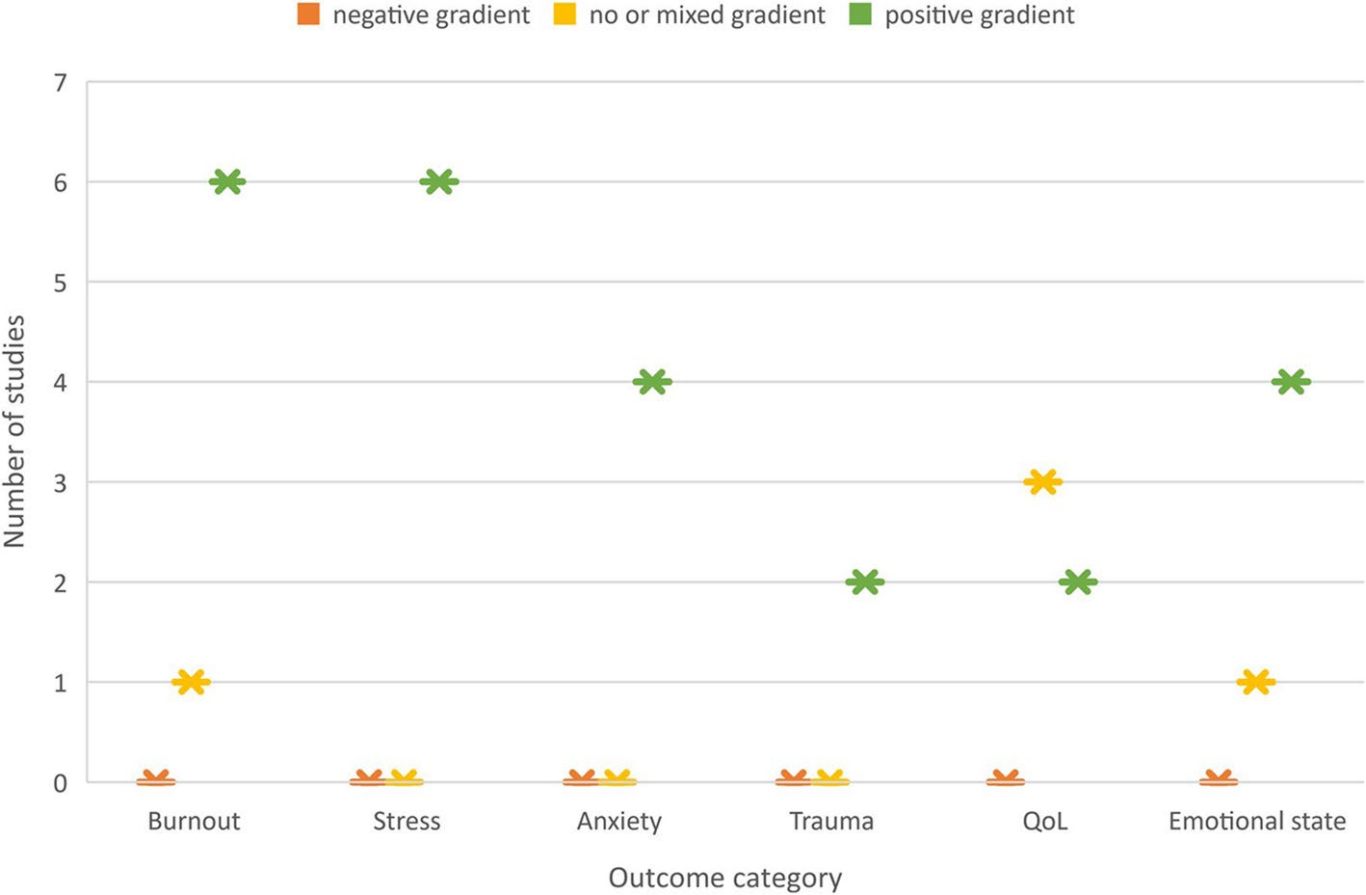
Harvest plot showing estimated direction of effect

Whilst it was not possible to pool effect due to heterogeneity, vote counting suggested direction of effect. Figure 8 provides a summary of estimated direction of effect using vote counting. This should be consulted in conjunction with Table 4 and Figs. 4, 5, 6 and 7, as the summary combines heterogeneous studies of variable quality and certainty of evidence.

No studies found negative direction of effect (potential harm) following art therapy-based interventions across any of the conditions measured. All but one of seven studies measuring burnout (including three randomised controlled studies) found positive direction of effect overall, although the size of effect varied across burnout symptoms. All studies measuring stress found positive direction of effect. All studies measuring anxiety, (including two randomised controlled studies) found positive direction of effect and both studies measuring post traumatic recovery reported positive direction of effect. All studies measuring an improvement in emotional state found positive direction of effect, although Mercer [37] reported mixed effects with positive change in negative affect but no change in positive affect. Studies measuring quality of life had mixed results, with 60% reporting no change and 40% reporting positive change. Ho [17] found medium effects using the EuroQoL quality of life measure, whilst Huet [38, 34] and Kometiani [30] reported no change in Professional Quality of Life (ProQOL) scores. However, the data were not available to calculate effect in these studies. Both Huet [38, 34] and Kometiani [30] reported discrepancy between quantitative and qualitative outcomes relating to Professional Quality of Life.

### Synthesised qualitative findings

**Fig. 9:**
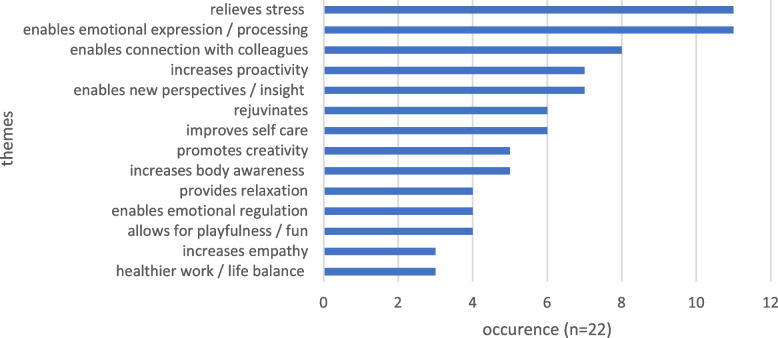
Qualitative themes of perceived effect across studies

Fourteen themes were identified following a content analysis of qualitative data extracted from 22 studies Fig. 9 shows the prevalence of themes of perceived effect across studies.

#### Relieves stress

Half of studies using qualitative methods reported stress reduction or an ability to better manage stress following the art therapy-based intervention. This perceived effect was identified both by participants and practitioners delivering the intervention. For example, an oncology nurse at a Korean hospital reported that when she threw clay her stress seemed to fly away as well [39] and a palliative care social worker in Hong Kong shared “*I feel now (after reflective art-making) I can actually cope with stress better*” [17]. Participant experience was mirrored in practitioner reports: “*all of the participants reflected how the art therapy group helped manage stressors at both home and work*” [30] p. 124 and “*Overall, it seemed that the drawing process helped the participants to better visualize their stressors, more easily focus upon them, and more readily transform them into positive emotions*.” [37], p. 147.

#### Expression and processing of emotions

Half of relevant studies reported the intervention facilitated expression and / or processing of emotions. For example, a HCW in an out-patient setting in Brazil explained: “…*there (in the workshop) you could get out what you were feeling by doing the activities. You did it and then you had to comment on what you did. So that was already a way for you to vent what you were feeling*” [33] p. 6. Analysis of participant comments (from interviews and group discussion) revealed that HCWs in the UK found viewing and discussing artworks enabled them to communicate thoughts and feelings otherwise difficult to verbalise [38].

#### Enables connection with colleagues

Eight studies reported an effect of the intervention to be positive connection with colleagues. For example, an end-of-life HCW in Hong Kong commented: ‘*Perhaps one of the greatest benefits was the realization, ‘‘I’m not alone, other professionals also face the things that I’m encountering’’*’ [40] p. 49. This was mirrored in survey responses of oncology doctors at a UK hospital who commented they particularly liked group work and sharing experiences of patients and colleagues within a safe environment [29]. South African hospice workers also valued bonding with the group. Listening to others helped them realize they were not alone in their experiences and they drew inspiration from each other’s stories. [41]

#### New perspectives and personal insight

Seven studies reported increased personal insight or changed perspective. Analysis of participant interviews found that new perspectives were developed through art [38]. Hsu [42] and Drapeau [32] found that engagement with Zentangle art and response art respectively cultivated self-awareness and promoted insight.

#### Proactivity and making positive changes

Seven studies reported proactivity, improved problem solving or feeling empowered to make positive changes. A paediatric HCW reported she would “*creatively look for new solutions*”, and that *“[the group] made me try some new things…which is a confidence builder*” [30] p. 124. British HCWs made significant changes to their jobs to address work stress, attributing this at least partly to the art therapy-based group [38], and a Canadian study found a benefit of response art to be “*proactivity and improved mental and emotional availability*” [32] p. 6.

#### Rejuvenating

A rejuvenating effect was found across several studies. This was attributed to various causes. For example, a US-based nurse highlighted the rejuvenating effect of reconnecting with her inner playfulness: “*I was able to come into contact once again with the inner playful side of me that I had kept buried and it felt very rejuvenating. This helped me to understand how I had experienced some symptoms of burnout from all the pressures life brings when you don't allow yourself to stay in balance*” [43] p. 64. A HCW in a Brazilian out-patient setting commented: “… *it is a break (from work) where you can replenish your energies*” [33] p. 5.

#### Improves self-care

Several studies noted a positively changed attitude towards self-care. This was often attributed to improved personal insight. For example, a US-based nurse noted: “*The mandala creation was a big step for me in realizing that I have permission and an obligation to nurture my own needs in order to be better equipped for others*” [43] p.64. Similarly, a physician working in Hong Kong reflected: “*While I want to hold on to the hands of the vulnerable, I also recognize that I myself am a vulnerable being who needs love and support”* [17] p. 8. A UK-based doctor found art making a helpful method of self-care: “*I wasn’t sure what to expect, really wasn’t sure if it would help. Amazed – I have actually painted at home after a bad day at work, using my son’s paints, and it helped*” [29] p. 16.

Other less frequently distributed themes included relaxation, emotional regulation, healthier work / life balance, increased empathy towards patients, creativity, and body awareness. Whilst these themes don’t explicitly describe effect in relation to burnout or psychosocial distress, they are linked to improvement in these areas of interest within the contexts of the relevant studies and help to explain change. For example, feeling more creative was linked to improved confidence [30, 40], personal balance [30], proactive solution seeking [18, 30], and self-care [41]. Increased body awareness and mind / body connection was considered helpful in recognising and responding to signs of burnout and in facilitating emotional regulation. For example, following a mandala workshop, a nurse reflected: “*I am much more aware of my body and how I feel now so that I can recognize the warning signs*” [43] p. 64. Response art participants reported “*A sensory and visceral experience that facilitates emotional digestion and regulation*” [32] p. 8.

#### Practitioner / participant report of perceived risk or harm

Within the literature reviewed no harms were reported. Apart one RCT [16], studies did not report checking for adverse events. However, several authors considered potential risk following analysis of qualitative data. Two studies [38, 18] found that some participants experienced art making to be initially intimidating but their initial fears were soon alleviated. In Potash’s 2014 Hong Kong-based study, a hospice worker noted finding the challenge of making art in response to emotional material upsetting at first, but this too was resolved, as “she was able to observe her anxiety and channel it into meaningful expression” [31]. One nurse from a Korean hospital felt it was difficult to express freely as group members were from the same hospital. However, other participants found sharing with colleagues to be stress reducing [39]. Following analysis of qualitative data, a Korean study with newly hired nurses found it was possible to increase stress at the beginning of treatment with participants potentially being uncomfortable with their task and afraid of being compared to others [26].

### Synthesis of qualitative and quantitative findings

The quantitative and qualitative syntheses complemented one another overall, despite two mixed methods studies [30, 34, 38] reporting contradiction between their quantitative and qualitative findings. Qualitative findings generally helped to explain quantitative results, although there were gaps in linking the two to report on mechanisms of change. It was necessary therefore to refer to the broader literature to flesh out explanations.

The most commonly occurring themes of perceived effect derived through synthesis of qualitative data were *stress reduction* and the *expression and processing of emotions*. These findings resonated with the quantitative data which showed reduced stress and burnout scores. Whilst *expression and processing of emotion* was not specifically measured quantitively, a strong correlation between emotional processing and two core burnout symptoms: emotional exhaustion and cynicism, has been demonstrated in neurophysiological and neuroimaging research [44]. As impairments in cognitive and emotional processing are evident in individuals suffering from burnout, improved emotional processing could be seen to indicate an improvement in burnout symptoms.

The second most common theme was *enables connection with colleagues*. As with *emotional expression and processing*, this theme is not named as one of the conditions measured quantitatively but complements findings by helping to explain them. Feeling connected to others can lower levels of anxiety [45]. In addition, feeling connected to and strengthening bonds with colleagues can have a buffering effect when exposed to occupational stressors [46]. Therefore, the function of the interventions to facilitate meaningful collegiate connection could help to explain the reduction in anxiety seen consistently across studies measuring this condition. Relational support has also been found to be an enabler of post traumatic growth. Findings from a systematic review of factors associated with post traumatic growth found relational support from other HCWs to be an even stronger indicator than support from family and friends [47].

The themes *New perspectives and personal insight* and *Proactivity and making positive changes* overlap as making changes was attributed to the acquisition of new insight by some authors [38]. There was additional co-occurrence of *enables creativity* which was seen as both a vehicle for gaining new perspectives and a catalyst for proactive solution seeking [18, 30, 38]. These co-occurring themes indicate wisdom (an enabler of post traumatic growth [47]), empowerment to transform circumstances (such as work roles [38]), and changed behaviours contributing to burnout (such as poor boundary setting [41]). However, the relationship between gaining new insight and behavioural change was not fully explored in studies. Similarly, the relationship between feeling more creative and feeling empowered to make positive changes was underdeveloped.

*Rejuvenating* and *improves self-care* themes of effect help to explain positive change seen in the emotional exhaustion burnout subscale across quantitative studies. The metaphor of an ‘empty tank’ is often used to describe burnout. Feeling replenished and rejuvenated by engagement with an art therapy-based intervention and being able to maintain that through improved selfcare behaviours could refill the tank. Supporting this hypothesis, Ho [17] found positive direction of effect for burnout was maintained (and in fact increased) 12-weeks post intervention.

The qualitative theme *of increased empathy towards patients* was less frequently distributed, appearing in three studies. Quantitative studies reporting on the discrete burnout subscales found a modest or insignificant reduction in cynicism. Three studies (including an RCT and pilot RCT) found this to be the burnout symptom with the smallest effect. The complexity of HCWs relationships to empathy is hinted at within qualitative data. For example: “*The lessons on empathy and ramifications of snap judgements that I have picked up are invaluable to my sense of self as a compassionate physician and have provided me a unique opportunity to consolidate a very complicated topic (how to maintain compassion while avoiding fatigue and burnout) into a manageable framework”* ([48]p. 103). However, potential reasons for insignificant change in relation to this subscale of burnout on the Maslach Burnout Inventory (MBI) warrant further exploration.

Two studies [34, 38, 30] found a discrepancy between quantitative Professional Quality of Life Scale (ProQOL) scores and qualitative data from group discussion and / individual interviews. Participants of both studies had sub-clinical quantitative scores at baseline, however qualitative data pointed to high levels of work stress for several participants at baseline followed by stress-reducing behaviours post intervention.

## Discussion

This review has found that a variety of art therapy-based methods are being used to address psychosocial distress in HCWs in a range of job roles and clinical contexts across the globe. Whilst most interventions were delivered by art therapists (either individually or alongside another professional) a small number were delivered by other HCWs such as a psychiatric nurse or a psychologist. Interventions were almost all provided in a group format and were most identified as “art therapy” or “art therapy-based” interventions. However, the frequency and duration of sessions, group size and therapeutic methods used varied, limiting scope to compare the relative effectiveness of different intervention characteristics and therapeutic methods, or to make firm recommendations for practice based on these. Whilst a handful of studies named a specific theoretical model of practice, such as Cognitive Behavioural Art Therapy [49] and Mindful-Compassion Art-Based Therapy [17], the majority did not. This is an area warranting attention in the future development of art therapy interventional studies.

Art therapy-based interventions have been found broadly acceptable by a range of HCWs across international contexts [16, 33]. However, differences in attitude towards sharing emotional material with colleagues have been reported [39, 34]. Further culturally sensitive research into participant experience and therapeutic approaches in relation to this is warranted. Studies are clustered primarily within Asia, North America and Europe, and within high income countries. Single studies have emerged from high-middle income countries in Africa and South America. Further research globally into the use of art therapy-based methods with HCWs in low to middle- and low-income countries would provide helpful insight into feasibility and breadth of application.

Whilst findings of this review indicate burgeoning research interest into the application of art therapy-based methods for HCWs, the preliminary nature of many studies and low number of RCTs indicate that research in this area is under-developed. The lack of established, high-quality research could point to broader issues within the art therapy profession. Research methodologies have not historically formed part of or been taught at a high level on art therapy training courses [50] and there has been some historic resistance to quantifying art therapy within the profession [51]. The review also uncovered a lack of arts-based and multimodal research methodologies and methods, arguably well suited to explore art therapy, and despite increasing attention these methods are receiving in health and social sciences research. The two pilot RCTs employing an active comparative condition (colouring [18] and guided visualisation [52]) both reported an exploratory purpose, with intention to examine both conditions, rather than use one condition as a control and the other as the intervention as in standard RCT design. Both studies found similar effects across both conditions.

Whilst qualitative data helped to explain quantitative findings, there were some gaps in identification of mechanisms of change. For example, an area warranting further explanation was the role of the body / mind aspect of art therapy-based interventions. *Increased body awareness* emerged as a theme in five studies but was reported as a perceived effect by participants only. Whilst the use of biomarkers to measure stress in two studies [18, 49] may indicate movement towards consideration of interlinked physiological and psychological effects, practitioner explanation of how the body / mind aspect of art therapy-based methods might function as a mechanism of change is notably absent.

Considering the systemic and multi-factorial problem of burnout and psychosocial distress in HCWs, these aspects were under-explored in most studies. Rigorous qualitative methods such as ethnographic research could be applied to contribute valuable knowledge in this area.

### Limitations

This systematic review has several limitations. Broad search terms and generous selection criteria, implemented due to an anticipated dearth of research, resulted in inclusion of a broad range of study designs and therapeutic methods. Whist this enabled a fuller understanding of the breadth of practice in the field, it did not support an empirical analysis of effect. For example, reporting was based largely on expert opinion in some case studies which were included but provided limited contribution to analysis. Despite broad inclusion criteria, this review retrieved only four studies using a randomised controlled methodology. This factor, along with heterogeneity in relation to outcome measures and design meant it was not possible to conduct a meta-analysis or to pool effect.

### Recommendations

Based on findings from this review the following recommendations are made for future research into the application of art therapy-based methods for HCW support:

- The inclusion of active control conditions in future RCT designs.
- The use of screening and baseline cut-off scores for inclusion in research studies may facilitate clearer clinical outcomes.
- The collection of follow-up outcomes measures and surveys would contribute to better understanding of potential longevity of effects.
- Fuller explanation of the relationship between quantitative and qualitative data in mixed methods studies to develop understanding of therapeutic mechanisms of change and provide indicatory recommendations for future practice and research.
- Rigorous qualitative research aimed at understanding systemic and intersectional factors.
- More widespread reporting of adverse or unexpected events during art therapy to inform intervention development and lower risk of bias.
- Clearer reporting of participant demographic characteristics to improve understanding of what is helpful for whom to target services effectively.
- Clearer intervention reporting including adherence data to inform intervention development and lower risk of bias.
- Research into how the risk of personal exposure might be mitigated through intervention design and delivery considerations such as therapeutic model and number of sessions.
- A more coordinated approach by researchers in the field to address widespread heterogeneity and enable the emergence of a robust evidence base.

## Conclusion

This systematic review has shown that global research into the use of art therapy-based methods to address burnout and psychosocial distress in HCWs is growing. A variety of interventions, applied across a range of clinical and international contexts were identified, highlighting wide-ranging and adaptable use of art therapy-based methods for HCWs. Whilst a meta-analysis was not possible due to heterogeneity of study design, estimated effects were reviewed, revealing a coherent picture of positive direction of effect with a high certainty of evidence for several conditions. A content analysis of qualitative data of perceived effect from both participants’ and therapists’ perspectives complemented quantitative findings and began to explain mechanisms of change. Studies were variable in methodological rigour and, whilst further high-quality evidence such as randomised controlled trials is needed to build a robust evidence base, findings already suggest that art therapy-based methods should be strongly considered as an acceptable and effective means of addressing emotional exhaustion (burnout) and psychosocial distress in HCWs.

### Supplementary Information

**Additional file 1.**

## Data Availability

Effect sizes were calculated using the data available in the included studies.

## Footnotes

1 This was not possible for effect size estimates but was the case for direction of effect in for Moss et al., 2022.
