# Supplementary material for: Art therapy-based interventions to address burnout and psychosocial distress in healthcare workers—a systematic review
Source: BMC Health Serv Res. 2023 Oct 4;23:1059. doi: 10.1186/s12913-023-09958-8 (PMC10552408; doi:10.1186/s12913-023-09958-8)
Supplement: Supplementary file 1 — Additional file 1. [file 12913_2023_9958_MOESM1_ESM.docx]

| **Paper** | **Name of Intervention** | **Why this intervention?** | **What materials and procedures supported it?** | **Who provided it?** | **How was it provided?**  **(e.g. group or individual)** | **Where was it delivered?**  **(e.g. country and setting)** | **When and How Much?**  **(Number, frequency, duration of sessions)** | **Has the intervention been tailored for a particular cohort?** | **Were any modifications made during the study?** | **How well was it delivered? (e.g. adherence, fidelity, evaluation)** |
| --- | --- | --- | --- | --- | --- | --- | --- | --- | --- | --- |
| **두은영 2018**  *Effects of Group Art and Music Therapy in Newly Hired Nurses: A Mixed Method Study* | Art Therapy | 1.Pre-existing studies show art therapy can improve self-confidence and self-esteem by supporting emotional expression in healthcare settings.  2. Previous findings that art therapy can reduce job stress in nurses.  3. Urgent need to find a solution to high rate of turnover in newly qualified nurses, believed to be due to job stress. | art making on a theme and with specific directives (including drawing and painting Korean orchids with eyes closed, focusing on feelings and sensations) psychoeducation (group discussion about how to relieve stress in daily life). relaxation exercise (involving drawing a Korean orchid in the air whilst relaxing their bodies and listening to music), sharing and reflective discussion | Art Therapist | Groups of 10 -12 | A general hospital in Korea | **Two 90-minute sessions**  **(Art therapy and music therapy were conducted consecutively with an interval of one week)** | The intervention was structured for the needs of the cohort and their working context | **Six nurses wanted to have additional art therapy, so two additional 90-minute art therapy sessions were delivered.** | Intervention was evaluated by participants |
| **Belfiore 1994**  *The group takes care of itself: Art therapy to prevent burnout* | Art Therapy | Art therapy’s capacity to enable participants to find new ways to express their experiences on deep level. | art making on a theme/ specific directives, free art making, group art making (working on same output), psychoeducation, sharing / reflective discussion, guided imagery | Art Therapist | Group of 8 | A community hospice care setting in Italy | **Twenty 3-hour sessions delivered weekly** | Generally adapted to needs of the cohort | Themes and creative projects were flexibly developed in relation to the needs of the group. | Not reported |
| **Depret 2020**  *Health and well-being: art therapy for health professionals working in outpatient care settings* | Art Therapy | 1.Growing global interest in the use of the arts in health care settings  2. World Health Organisation (WHO) report of emerging evidence for the therapeutic use of art to improve wellbeing in a range of populations  3. The growing need to find HCW support solutions | art making on predetermined themes, sharing and reflective discussion, use of narrated stories | Psychologist and an ‘Art Therapy Specialist’ | Groups of 6 | Outpatient philanthropic organisation, São Paulo, Brazil | **Six 1.5-hour sessions delivered fortnightly** | The intervention was structured for the needs of the cohort and their working context | None specified | Not reported |
| **Drapeau 2022**  *Beyond art expression: Understanding participants’ experience and outcomes of a vicarious trauma and response art workshop* | Response Art | 1.Based on art therapy literature describing the benefits of response art (RA) to help art therapists express and process clinical work.  2. Historic documentation of the benefits of art therapy for processing trauma. | psychoeducation (PowerPoint presentation on vicarious trauma and theoretical basis of response art); free art making, sharing and reflective discussion, provision of Response Art kits and Self-reflection guides with training how to use them | Art Therapist | Group workshop followed by individual independent RA at home | Canada. Workshop setting not specified | **One three-hour session (with individual follow up at home using the RA kit)** | The intervention was structured for the needs of the cohort and their working context | None specified | All participants attended the workshop. 5 out of 7 reported using RA at follow up |
| **Duffy-Randall 2006**  *Mandala: a way of learning transpersonal nursing* | Mandala making | The workshop formed part of a retreat aimed at replenishing nurses through the application of transpersonal nursing theory and practice.  The inclusion of mandala making was due to the historic use of the artform for personal and spiritual discovery and growth. | art making on a theme/ specific directive, walking meditation, sharing and reflective discussion | Psychiatric Clinical Nurse Specialist | Groups of 5-7 participants | Historic retreat centre in Texas, United States. Mandala making took place in a multi-level spiral – shaped tower overlooking countryside. | **One 90-minute session** | Intervention developed for the cohort based on a transpersonal nursing framework. | Not discussed | Not discussed |
| **Ho et al 2021**  *A Novel Mindful-Compassion Art-Based Therapy for Reducing Burnout and Promoting Resilience Among Healthcare Workers: Findings from a Waitlist Randomized Control Trial* | Mindful-Compassion Art-Based Therapy (MCAT) | Recent burgeoning of literature on the effects of mindfulness practice and art-based therapy to reduce stress and improve mental health. | art making on a theme (including Mandala making), group art making (including a mural), mindfulness-based stress reduction exercises, psychoeducation, sharing, reflective discussion, reflective writing, guided imagery | Two MCAT therapists including one accredited art therapist and one clinical researcher trained in mindfulness-based stress reduction | Groups of 9-10 participants | Singapore. Home hospice context. Setting not specified. | 6 weekly sessions of 3 hours each. | Structured intervention protocol designed to fit the context. | Not discussed | Group Attendance not detailed. However all participants completed the study with no attrition throughout the research period |
| **Hsu 2021**  *Effects of Zentangle art workplace health promotion activities on rural healthcare workers* | Zentangle art-based intervention | 1.Historic evidence of the capacity for art therapy to reduce mental distress in HCWs  2. Literature on the calming effect Zentangle Art which harnesses meditation through art making | Art making with specific directives and relaxation exercise using the Zentangle method, participants given art materials to take home and use in their own time | Certified Zen-tangle Teachers (CZT®) | Group of 20 | Hospital in rural Taiwan | **Single 4-hour session** | Structured intervention protocol designed to fit the context. | None identified | Adherence to protocol not reported. Attendance not reported. Effect of intervention formally evaluated. |
| **Huet 2011**  *Art therapy-based organisational consultancy: a session at Tate Britain* | Art therapy-based organisational consultancy | 1. Promising initial reports of the use of arts – based methods to support palliative care staff.  2. Wish to develop art therapy literature discussing application of art therapy to work with organisations.  3. Interest in finding ways of bringing creativity into organisational consultancy | art viewing, art making with specific directives, sharing and reflective discussion | Art Therapist and curator | Group of 2 | Art gallery (after hours), London, UK | **One 2-hour session** | Structured intervention protocol designed to fit the context. | 10 signed up but only 2 attended on the day which led to adapted delivery | Limitations of the intervention in relation to the impact of low attendance was reported. |
| **Huet 2016**  *Art therapy-based groups for work-related stress with staff in health and social care: An exploratory study* | Art therapy-based groups | 1.Growing interest in the capacity of the arts to contribute to health and social care workers’ wellbeing is not sufficiently supported by robust research.  2. The intervention was designed to develop knowledge of active processes of art therapy-based groups with health and social care workers to reduce work-stress. | art viewing, art making with specific directives, sharing and reflective discussion | Art Therapist | Groups of 4 - 6 | Hospice and other health and social care work settings, United Kingdom. | **Three 90-minute sessions at monthly intervals** | Structured intervention protocol designed to fit the context. | None identified | Intervention adherence not reported.  Attendance information not provided. |
| **Huet 2017**  *Case study of an art therapy-based group for work-related stress with hospice staff* | Art therapy-based groups | 1.Previous case studies on the use of art therapy with hospice staff have reported wellbeing benefits.  2. Conducted as part of a larger research project to develop knowledge of active processes of art therapy-based groups with health and social care workers. | art viewing, art making with specific directives, sharing and reflective discussion | Art Therapist | Group of 6 | Hospice setting, United Kingdom. | **Three 90-minute sessions at monthly intervals** | Structured intervention protocol designed to fit the context. | None identified | Intervention adherence not reported. |
| **Huss 2014**  *Visually transforming artwork and guided imagery as a way to reduce work related stress: A quantitative pilot study* | Art therapy workshop  (Compared to guided imagery workshop) | 1.Historic use in healthcare to address occupational stress. 2.Previous findings on the use of creative transformation of disturbing visual images warranted further elaboration. | art making with directives (creatively transform a stressful mental image), psychoeducation, guided imagery | not specified | Groups of 17 -18 | Ben-Gurion University of the Negev, Israel. Department of social work | **5 hours over two consecutive days** | Structured intervention protocol designed to fit the context. | None identified | Intervention adherence not reported. Attendance information not provided. |
| ***Jang 2015***  *The effects of a group art therapy on job stress and burnout among clinical nurses in oncology units* | Group art therapy | 1. Historic use of group art therapy as treatment for a range of patient populations.  2.Findings from recent studies using group art therapy with office workers showing positive effect on job stress factors.  3.Capacity to encourage emotional expression through art making.  4. Capacity of group art therapy to develop interpersonal relationships.  5. Therapeutic effect which cannot be expected in individual therapy | art making on a theme, sharing and reflective discussion, using art materials in a physical manner (tearing and throwing), group trust building exercises | Art Therapist | Groups of 14 - 15 | Training centre in a University Hospital in Korea | **8 weekly sessions of 90 minutes** | Structured intervention protocol designed to fit the context.  Groups delivered outside of working hours due to the difficulty adjusting shift work for the nurses. | None identified | Intervention adherence not reported. Attendance information not provided, although participant feedback indicates some non-attendance due to being too busy and not being able to finish work in time. |
| **Kaimal 2019**  *Outcomes of art therapy and coloring for professional and informal caregivers of patients in a radiation oncology unit: A mixed methods pilot study* | Open studio art therapy and colouring | 1.Previous findings regarding the potential of art and art therapy – based methods to helpfully contribute to HCW psychosocial support.  2. Desire to build on existing research which is limited | Open studio art therapy condition: free art making, art therapist art making alongside the participant, reflective discussion (following the participant’s lead), guidance on art making process offered  Colouring condition: Colouring of a page selected from a pack using a choice of coloured pencils or markers. | Art Therapist (for the open studio) art therapy session.  No facilitator for colouring (researcher outside the door) | Individual | Interventions took place in a side room outside the radiation oncology unit of a hospital in the United States. | **Single 45-minute session.** | Elements of intervention delivery (such as location) tailored to the fit the context. | None identified | Intervention adherence not reported.  Attendance information not provided.  Intervention effects formally evaluated. |
| **Kinsella Frost 2019**  *Art in debrief: a small-scale three-step narrative inquiry into the use of art to facilitate emotional debriefing for undergraduate nurses* | Art-based debrief | 1.Personal nursing and teaching experiences of the value of art and creativity in processing thoughts and articulating emotions.  2.The identification of a gap in debriefing for trainee nurses based on a review of the literature. | art making on a theme and with specific directives, reflective discussion (with researcher), art viewing, reflective writing | Nursing lecturer | **Not specified** | Educational setting where undergraduate nursing students spend 50% of their programme in clinical practice. Space not specified. | **Not specified** | **Not specified** | None identified | Intervention adherence not reported.  Three out of six initial volunteers participated due to time commitment required.  Thematic analysis of participant reflections on the process. |
| **Kometiani 2017**  *Creating a vital healing community: A pilot study of an art therapy employee support group at a pediatric hospital* | Art therapy support group | 1.Historic studies demonstrating the use of art therapy to address stress and associated psychosocial issues for HCWs  2. Historic studies demonstrating the use of art therapy to support team building | Procedures: art making with directives (e.g. worry doll making and deconstructing / embellishing a book), sharing and reflective discussion, creating vision boards, setting goals and intentions. Materials included collage materials, stamps and stencils | Art Therapist | Group of 13 | Paediatric hospital, United States (part of an ongoing staff support group) | **3 monthly sessions of 45-60 minutes** | Intervention designed to fit the context with art materials and creative processes chosen to be accessible regardless previous experience of art making . | None identified | Adherence not reported. Attendance varied, with full attendance in the final session. |
| **Mercer 2010**  *Visual journaling: An intervention to influence stress, anxiety and affect levels in medical students* | Visual journaling | 1.Previous studies finding journal writing to be effective at reducing stress and anxiety in medical students  2. Previous studies finding art making to be stress reducing and emotionally satisfying | art making on a theme / with specific directives, relaxation exercise (guided imagery whilst focusing on breathing), creative and reflective writing, visual journaling | Not specified | Group of 10 | Eastern Virginia Medical School, United States | **2 sessions with a two – week interval. Duration of sessions not specified.** | Intervention designed to fit the context. | Recruitment issues resulted in less time to deliver the group intervention than initially planned. | Intervention adherence and attendance not reported. Intervention formally evaluated. |
| **Moss 2022**  *The Effect of Creative Arts Therapy on Psychological Distress in Healthcare Professionals* | Creative Arts Therapy | 1.Historic literature on the potential for the creative arts therapies to help HCWs develop coping strategies.  2. Historic literature on the capacity of creative arts to elicit emotional expression and support emotional processing. | Creation of journal pages about experiences and emotions. Group sharing.  Materials included a blank journal and a range of art materials including oil pastels and watercolour paints.  (Dance movement, creative writing and music groups were alternative options) | Not specified, although the intervention was developed by the hospital’s creative arts therapists. | Groups (size not specified) | Several Denver hospitals, USA | 12 weekly sessions of 90 minutes | Intervention designed to fit the context. | None identified | Attendance was monitored and reported. Median attendance:  9.5 of 12 sessions. Further adherence to the intervention not discussed. |
| **Nainis 2005**  *Art therapy with an oncology care team* | Art therapy | 1. Historic literature on the use of art therapy to address grief and emotional stress.  2. Historic literature on the helpful effects of response art for emotional processing in nurses | Art making on a theme / with specific directives, group art making (group quilt).  Materials included paint. | Psychosocial care team including social workers, a nurse, a recreation therapist and an art therapist | Groups of 3-5 | Oncology unit, large Midwest academic hospital, United States | Single 2-hour session | Intervention designed to fit the oncology unit context. | None identified | Adherence and attendance not reported. |
| **Patel 2021**  *Overview of an emergent, arts-based resiliency curriculum to mitigate medical trainee burnout* | Arts-based resiliency curriculum | 1.Historic use of arts in health education.  2.The programme builds on joint art museum / medical education programmes aimed at honing medical students’ analytical and diagnostic skills.  3.Previous studies using art to develop physician empathy | art making on a theme / with specific directives, group art making (working on same output), sharing / reflective discussion, art viewing, creative / reflective writing, tableau vivant | Arts Educator and Medical school faculty member | Groups of 15 - 20 | The University of Tennessee College of Medicine. Compulsory training module. | Monthly sessions of 2.5 hours. No cap on number sessions attended. (Trainees spent up to two years at the college and could attend as many sessions as they chose). | Intervention designed to fit medical trainee’s context | None identified | Adherence not reported. Attendance was monitored and the intervention was evaluated. |
| **Potash 2014**  *Can art therapy reduce death anxiety and burnout in end-of-life care workers? A quasi-experimental study* | Art therapy | 1.Documenatation in the literature of art therapists using art therapy methods to support end of life workers with grief and emotional processing.  2. Historic literature on the use of art to facilitate emotional processing  3. Historic literature on the role of supervision in support of end-of-life workers. | art making on a theme / with specific directives (e.g. visually representing stress then transforming it); relaxation exercise (breathing), sharing / reflective discussion, supplementary reflective writing | Art therapist  (Nurse consultants and counselling psychologists delivered comparator skills-based sessions) | Groups of up to 25 | Hong Kong end of life healthcare context | Art therapy – bases group: Six weekly 3-hour sessions  Skills - based group: Three daily 6-hour sessions | Intervention protocol designed for the context | None identified | Adherence and attendance not reported. Programme was formally evaluated. |
| **Potash 2015**  *A Model for Art Therapy–Based Supervision for End-of-Life Care Workers in Hong Kong* | Art Therapy–Based Supervision | 1.Historic reporting of art therapy reducing burnout in palliative care workers and mitigating stress in oncology nurses.  2. Previously documented use of art therapy to  enable self-care and enable expression of grief in relation to patient deaths.  3. Historic reporting of mandala making being energising and centring for palliative care workers. | art making on a theme / with specific directives (e.g. mandala making), group art making (mural) mindfulness -based exercise including grounding and guided imagery, sharing / reflective discussion, reflective writing, personal reflection | Professional title of facilitator not specified | Groups of 23 - 25 | End of life care context, Hong Kong. Group setting  not specified | Six sessions of 2 – 2.5 hours | Intervention protocol designed for the context | Not discussed | Adherence to the intervention not discussed. Intervention was evaluated through participant feedback. |
| **Potash 2016**  *Medical student mandala making for holistic well-being* | Mandala making | Mandala making was selected based on previous cross-discipline findings in relation to their usefulness reducing anxiety and depression and enhancing self-awareness. | art-making workshop during which mandalas based on current emotional state were created. Followed by reflective writing and discussion. | Art Therapist | Group (size not specified) | Art studio at a holistic health research centre based at Hong Kong University | Singular session of 2.5 hours | The intervention was designed for the context | Not discussed | Adherence to the intervention not discussed. Evaluation of methods not discussed. |
| **Salzano 2013**  *The effectiveness of a collaborative art-making task on reducing stress in hospice caregivers* | Collaborative art making | 1.Historic documentation of the use collaborative art therapy techniques with teams in oncology and palliative care settings. | art making on a theme and with specific directives, group art making (collaborative quilt), reflective discussion | Art Therapist / Researcher | Groups of 10 | Conference room in a hospice, United States | Control (staff meeting) followed by single intervention session of 45-50 minutes | A structured protocol was designed for the setting context. | Not discussed | Adherence was not formally evaluated. Attendance was reported (7 participants  excluded from data analyses  because they were not present for both the control and the experimental conditions). The intervention was formally evaluated. |
| **Tjasink 2019**  *Art therapy to reduce burnout in oncology and palliative care doctors: a pilot study* | Art therapy | 1.Historic and recent literature on the use of art therapy with HCWs  2.History of providing art therapy – based professional development workshops for hospital staff  3.Approached by staff for art therapy support | Art making on a theme and with specific directives, group art making (working on same output e.g. group clay sculpting), sharing and reflective discussion | Art Therapist | Groups of 2-8 participants | Hospital art therapy room and hospital meeting rooms based at a large teaching hospital in London, UK | Six weekly sessions of 90-120 minutes. | Intervention designed with input on from participants to fit practicalities of their working context. | Low number of participants in the second group led to more time for discussion with the art therapist, but less input from colleagues. | Adherence not formally evaluated. Mean attendance was 71.4%. Intervention formally evaluated. |
| **Van Westrhenena 2013**  *The experiences of professional hospice workers attending creative arts workshops in Gauteng* | Creative expressive art therapy | Creative debriefing process to facilitate expression and communication of thoughts and feelings. | Art making on a theme or with specific directives; relaxation exercises; sharing and reflective discussion; creative / reflective writing, massage, music making, soul cards, dancing, drama debriefing | Psychologist | individual and group: | Gauteng province, South Africa. Hospice context. (Workshop setting not specified) | 45-minute individual meeting followed by  four two-day workshops and  one three-day workshop | Tailored to cultural context | Not discussed | No evaluation of the intervention reported |
| **Visnola 2010**  *Effects of art therapy on stress and anxiety of employees* | Art Therapy | Historic findings relating to the effects of creative activities on cortisol levels. | art making on a theme / with specific directives (e.g. “tree of emotions”, free art making, group art making (e.g. group collages), psychoeducation, relaxation exercise (e.g. releasing areas of stress in the body) sharing / reflective discussion, painting whilst listening to music, homework of daily scribbles | Art Therapist | Groups (size not specified) | Latvia. Health context. (Workshop setting not specified) | Nine weekly 2-hour sessions | The intervention protocol was structured for the needs of the cohort and their working context | Not discussed | Adherence and attendance not reported. Effects of intervention formally evaluated. |
| **Yılmaz 2018**  *Effect of a nurse‐led intervention programme on professional quality of life and post‐traumatic growth in oncology nurses* | Nurse‐led intervention programme | The intervention was based literature about the benefits of emotional expression through art making, psychoeducation, support groups and counselling. | Mandala painting, psychoeducation (lecture, reading and a video demonstration), exercise and baksi dance.  Follow – up motivational messages were sent via text message. | Nurse | Group (size not specified) | Oncology unit, University Hospital in Izmir, Turkey | Two sessions and two follow - ups | Structured intervention protocol tailored to context. | Not discussed | Adherence not reported. No participants left the programme. |
